# Identification of an Ultra-Rare GLA Frameshift Variant in a South African Family With Hypertrophic Cardiomyopathy: A Case Report

**DOI:** 10.7759/cureus.79668

**Published:** 2025-02-26

**Authors:** Timothy F Spracklen, Polycarp Ndibangwi, Ntobeko A B. Ntusi, Sarah Kraus, Gasnat Shaboodien

**Affiliations:** 1 Department of Paediatrics and Child Health, University of Cape Town, Cape Town, ZAF; 2 Department of Medicine, Cape Heart Institute, University of Cape Town, Cape Town, ZAF; 3 Extramural Unit of Noncommunicable and Infectious Diseases, South African Medical Research Council, Cape Town, ZAF; 4 Division of Cardiology, Department of Medicine, University of Cape Town, Cape Town, ZAF

**Keywords:** clinical case report, fabry diseases, gla gene, hypertrophic cardiomyopathy (hcm), south africa

## Abstract

Fabry disease (FD) is an X-linked deficiency in glycosphingolipid metabolism caused by pathogenic variation in *GLA*. FD can mimic hypertrophic cardiomyopathy (HCM). Here, we present a South African patient of European ancestry with HCM where subsequent genetic analysis led to a diagnosis of FD. He was diagnosed with HCM at the age of 53 when he presented with new-onset atrial fibrillation (AF) and a left occipital cerebral infarction. He reported receiving treatment for recurrent pneumothoraxes in his mid-20s and suffered a transient ischemic attack (TIA) seven years prior to his diagnosis. During follow-up, he developed progressive chronotropic incompetence with AF and symptomatic bradycardia requiring pacing, as well as progressive dyspnea with obstructive lung disease, mild proteinuria with grade 1 chronic kidney disease, and peripheral neuropathy. Genetic research led to the identification of a pathogenic frameshift variant (*GLA* c.774_775delAC; p.Pro259ArgfsTer5) in the patient and his mother. This is an ultra-rare 2 bp pathogenic deletion in the causative gene for FD. Therefore, a diagnosis of FD was considered in this family and subsequently confirmed by an enzyme activity test. The proband was started on enzyme replacement therapy (ERT) to preserve kidney function and prevent other organ involvement, although it was not expected to reverse cardiac hypertrophy. This case demonstrates that non-cardiac disease may precede cardiac presentation in FD, emphasizing the importance of a detailed medical history in patients presenting with HCM. Testing for *GLA* variation should be considered in patients with similar phenotypic presentation, as diagnosis of HCM phenocopies such as FD can have important implications on treatment and management. Timely treatment of FD with ERT is crucial to prevent end-stage organ damage and preserve quality of life.

## Introduction

Hypertrophic cardiomyopathy (HCM) is one of the most common forms of cardiomyopathy, with a reported prevalence of one in 500 individuals [1]. HCM refers to the presence of increased left ventricular wall thickness or mass (with or without right ventricular hypertrophy) that is not solely due to abnormal loading conditions, leading to diastolic dysfunction and a high risk of ventricular arrhythmias and sudden cardiac death. Up to 50% of HCM cases are genetic with causative variants in sarcomeric genes [2]. Phenocopies of HCM include systemic metabolic conditions with cardiac involvement such as storage and infiltrative disorders that can cause left ventricular wall thickening unrelated to sarcomeric gene defects. Due to distinct mechanisms underlying HCM and its mimics, identifying phenocopies can have profound effects on treatment and management.

Fabry disease (FD) is a rare, progressive, and multisystem storage disorder caused by α-galactosidase A (*GLA*) gene variants leading to systemic globotriaosylceramide (Gb3) deposition. It typically includes renal, dermatological, ocular, cardiac, and neurological manifestations. Cardiac involvement can include progressive left ventricular hypertrophy (LVH) similar to HCM or myocardial fibrosis. Predominantly cardiac manifestations of FD, where LVH occurs without other organ involvement, may be mistaken for HCM [3,4]. FD may constitute an important contribution to HCM prevalence, particularly among mutation-negative patients. As a global enzyme deficiency, the management of FD differs vastly from standard HCM treatments, which include medications and interventions to preserve cardiac function. Although FD currently has no cure, enzyme replacement therapy (ERT), when started early enough, can slow down tissue damage and prevent the progression of symptoms to end-stage illness.

In this case report, we present a South African patient with HCM, in whom subsequent genetic analysis led to a diagnosis of FD. Through genetic research, we identified an ultra-rare pathogenic *GLA* frameshift variant that was considered the cause of his disease; subsequently, he was found to have other symptoms of FD and was started on ERT. This case demonstrates the importance of screening for *GLA* variants in patients with apparent HCM, as well as the role of research and humanitarian programs in clinical practice in resource-limited settings. These data have been presented in Timothy Spracklen's doctoral thesis.

## Case presentation

**Figure 1:**
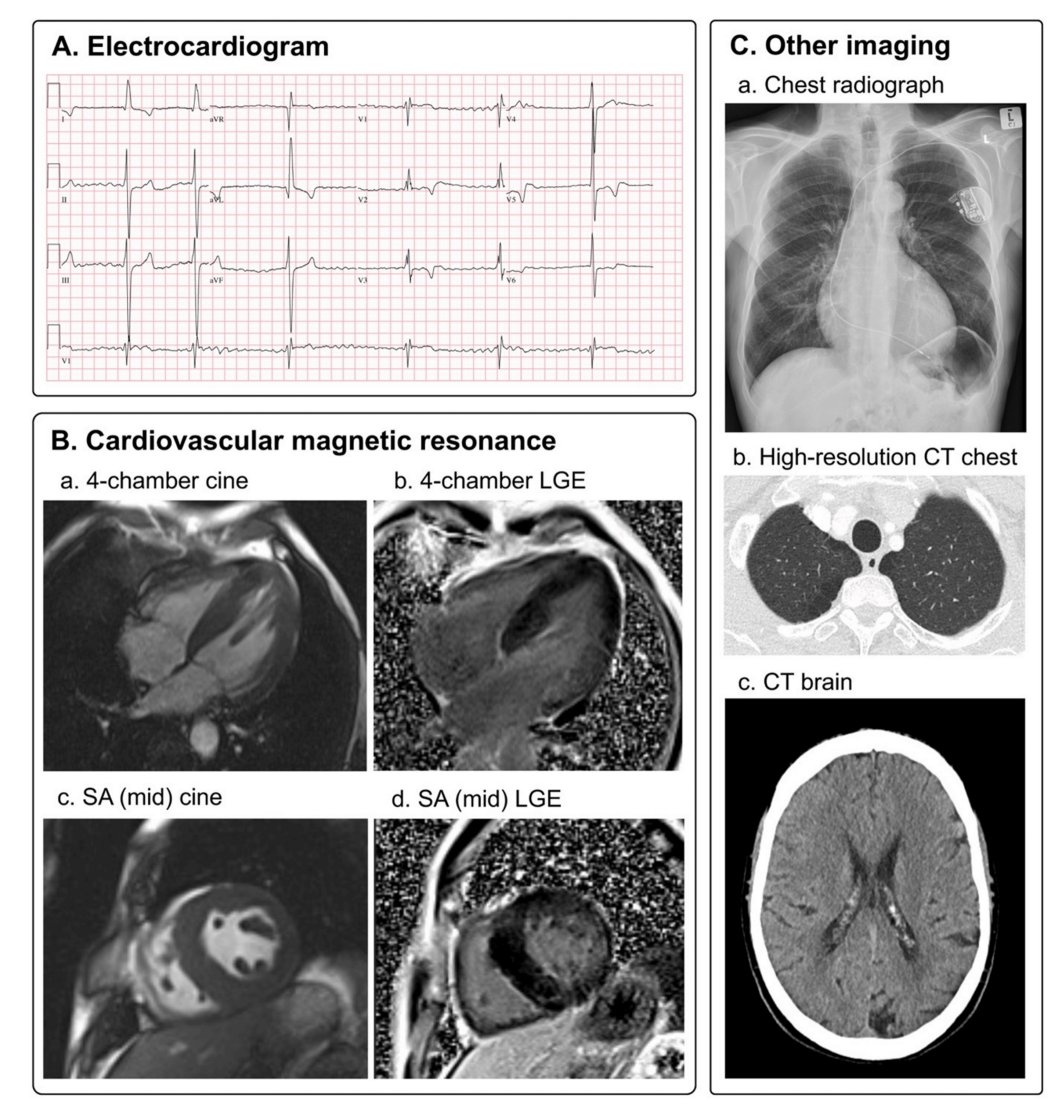
Clinical investigations in proband demonstrating multisystem FD (A) Electrocardiogram showing AF with bradycardia. (B) Cardiac magnetic resonance imaging showing concentric LVH (septum 24 mm) with patchy late gadolinium enhancement, particularly in the basal inferolateral left ventricular wall. (C) Chest radiograph (a), high-resolution chest CT (b) showing COPD with hyperinflation and emphysematous changes, and brain CT (c) showing left occipital encephalomalacia from a prior cerebral infarction. CT, computed tomography; LGE, late gadolinium enhancement; SA, short-axis; FD, Fabry disease; AF, atrial fibrillation; LVH, left ventricular hypertrophy; COPD, chronic obstructive airway disease

**Figure 2:**
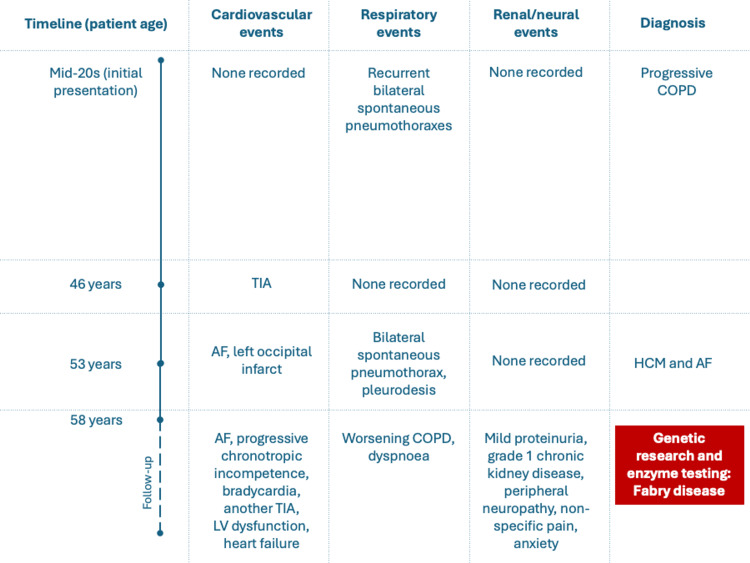
Timeline showing the progression of clinical events AF, atrial fibrillation; COPD, chronic obstructive airway disease; HCM, hypertrophic cardiomyopathy; LV, left ventricular; TIA, transient ischemic attack

A South African male of European ancestry and his mother were both diagnosed with HCM. The proband's diagnosis of HCM was at age 53 when he presented with new-onset atrial fibrillation (AF) and a left occipital cerebral infarction (Figure 1). On further inquiry, he had symptoms consistent with multisystem FD dating back to his mid-20s (Figure 2). He had initially presented with recurrent bilateral spontaneous pneumothoraxes, eventually requiring pleurodesis, and had been diagnosed with progressive chronic obstructive airway disease (COPD) of unknown etiology. He then presented with a transient ischemic attack (TIA) in his late 40s, for which he was investigated with no cardiovascular cause identified. Spontaneous pneumothoraxes are not often reported in FD; however, obstructive lung disease and TIAs are well described and represent early signs of the patient’s underlying FD.

Following the diagnosis of HCM, he developed progressive chronotropic incompetence with AF and symptomatic bradycardia requiring pacing. He reported progressive dyspnea due to worsening obstructive lung disease, in the absence of left ventricular outflow tract obstruction. He suffered another TIA while on anticoagulation, and later developed mild proteinuria with grade 1 chronic kidney disease, peripheral neuropathy with erectile dysfunction, and intermittent non-specific pain with worsening anxiety. Nine years after the diagnosis of HCM was made, he developed symptoms of heart failure with mild left ventricular dysfunction. These findings prompted genetic investigation through the African Cardiomyopathy and Myocarditis Registry Program (IMHOTEP), as genetic testing for cardiomyopathies is not readily available in the state service in South Africa.

Whole exome sequencing was conducted as described before [5]. Briefly, this entailed analysis of the proband’s genomic data for rare (<0.01), likely damaging variants in established cardiomyopathy and HCM phenocopy genes. Synonymous and intronic non-coding variants were excluded; only missense variants (CADD >20) and protein-truncating variants were considered. Genes outside the cardiomyopathy panel were considered as described before [5]; however, the genetic filtering led to the identification of a frameshift *GLA* variant, NM_000169.3:c.774_775delAC (p.Pro259ArgfsTer5), in the proband and his mother, which was considered the most likely cause of the observed phenotypes. This variant fulfilled the following American College of Medical Genetics (ACMG) criteria [6] for a pathogenic classification: it is a predicted null variant in a gene where the loss of function causes FD (PVS1, 8 points); it occurs in a mutation hotspot (PM1, 2 points); it is absent in population databases (PM2, 2 points); and it co-segregated with disease (PP1, 1 point). This variant is considered ultra-rare, as it is not present in any genetic population database (e.g., GnomAD), but has been reported in a single European patient with classical FD [7]. Enzyme activity was pathologically reduced in the proband (Table 1), confirming the diagnosis of FD.

**Table 1:** Clinical characteristics of the proband and family members AF, atrial fibrillation; HCM, hypertrophic cardiomyopathy; LGE, late gadolinium enhancement; LVH, left ventricular hypertrophy; ND, not done

Individual (age of onset in years)	Cardiac phenotype	Presenting cardiac symptoms	Electrocardiogram	Echocardiogram	Cardiac magnetic resonance imaging	Cardiac devices	Enzyme activity level
Proband (53)	HCM with AF	Palpitations	Sinus rhythm, LVH	Severe concentric LVH, diastolic dysfunction	Concentric LVH, extensive patchy mid-wall LGE	Permanent pacemaker	<2.8 µmol/L/h (normal range ≥15.3 µmol/L/h)
Proband's mother (68)	HCM with AF	Palpitations, syncope	Sinus bradycardia, sick sinus syndrome	Septal LVH	ND	Permanent pacemaker	ND
Proband's sister (NA)	At risk but clinically not affected	None	Sinus rhythm	Normal	ND	None	ND

Following confirmation of the FD diagnosis, our patient was started on ERT. A number of months after treatment initiation, he developed a mild-moderate infusion-associated reaction (IAR), which resolved after stopping the infusion and administering hydrocortisone. Severe type 1 hypersensitivity reactions to ERT are rare and occur in approximately 1% of patients [8]. IARs due to immunogenicity are more common, particularly in patients with little or no residual enzyme activity. Most patients develop antibodies to ERT, typically within three months of the first infusion. Over time, most seropositive patients demonstrate either a downward trend in antibody titer (40%), become tolerized (14%), or exhibit a plateau in antibody titer (35%) [8]. Patients with antibodies have a greater risk of developing IARs (defined as any related adverse event occurring on the infusion day); these patients should be treated with caution when administrating ERT, although an IAR is not a contraindication to continuing therapy and does not affect treatment response. ERT has been continued in our patient with no further reactions reported. While we do not anticipate the reversal of his cardiac disease due to the limited efficacy of ERT in clearing Gb3 in cardiomyocytes in later-stage disease, the aim of treatment is to preserve the function of organs more responsive to ERT such as the kidneys and lungs. ERT may also prevent further Gb3 accumulation in cardiomyocytes. After a year on ERT, there has been no worsening in our patient’s estimated glomerular filtration rate or proteinuria, his lung function and cardiac function are unchanged, and he reports improvement in fatigue and anxiety symptoms.

FD was confirmed in the proband's mother through genetic testing. She has a pacemaker in situ and continues to be monitored by her cardiologist, but declined ERT. The proband’s sister (asymptomatic at age 55) was counseled but declined genetic testing and clinical screening.

## Discussion

*GLA* variants and FD are an important cause of HCM [3], although the contribution of *GLA* variation to HCM in South Africa is currently unknown. To the best of our knowledge, this is the first report of a *GLA* mutation in a South African patient with previously unexplained LVH.

The pathogenic variant identified in this family was a 2 bp deletion resulting in possible nonsense-mediated decay. This means that, rather than exerting a dominant negative effect, this variant likely acts through the haploinsufficiency of the *GLA* gene. Both missense and null *GLA* variants have been described in HCM previously, spanning the length of the gene, indicating that cardiac manifestations are not restricted to a specific locus or variant type. Most similar to our case is the 2 bp deletion (c.718_719delAA) that was reported in an Italian HCM patient with AF and ventricular tachycardia [9]. This variant disrupts the same protein domain as in our case, and the mutation carriers were also noted to have arrhythmic phenotypes. Indeed, the cardiac variant of FD frequently includes arrhythmias such as short PR interval, AF, ventricular tachycardia, and sinus bradycardia [3,10,11]. Deficient or absent α-galactosidase A activity, caused by *GLA* mutations, results in the accumulation of Gb3 in the lysosomes of cells throughout the body [12]. As an X-linked disorder, males who inherit disease-causing *GLA* variants tend to have more severe presentation and/or earlier onset, as in our case, while carrier females can have a variable presentation due to X-inactivation. Cardiac hypertrophy and myocardial fibrosis in FD have been ascribed to the deposition of Gb3 in cardiomyocytes, while accumulation of Gb3 in conduction tissue can lead to arrhythmias or short PR intervals in patients [12].

The cardiac variant of FD is typically late-onset, presenting as unexplained LVH in middle-aged or older patients [13]. Although hypertrophy is usually symmetrical, asymmetrical septal hypertrophy has been observed in some cases of FD; in contrast, symmetrical hypertrophy is much rarer in HCM [10,13,14]. However, due to the slight overlap, the symmetry of ventricular hypertrophy itself is not enough of a distinguishing feature between HCM and FD. It is thought that FD may mimic HCM through *GLA* mutations that do not completely disrupt GLA protein function: individuals with "classical" FD have no detectable GLA activity, while the GLA protein in patients with the cardiac variant retains residual (approximately 10%) enzymatic activity [7,14,15]. Due to the vast clinical heterogeneity of FD, distinguishing it from HCM can be challenging without cardiac magnetic resonance T1 mapping, where native T1 values are lower in FD due to the presence of Gb3, which decreases T1 longitudinal relaxation time [16], or clinical genetic testing. Currently, the most sensitive diagnostic tests for FD include the enzyme activity assay or genetic testing, in which pathologic deficiency of the enzyme or gene can be detected [17]. Achieving a diagnosis of FD by signs or symptoms alone is challenging, especially in cases without a known family history of the disease.

This case demonstrates the importance of exploring the medical history of patients presenting with HCM in more detail, as non-cardiac disease may precede cardiac presentation. Identifying pathogenic *GLA* mutations in HCM patients has important implications for patient management, as in this case where identification of *GLA* c.774_775delAC in the proband led to confirmation of FD by enzyme activity test, followed by the initiation of ERT. ERT is a treatment in which deficient α-galactosidase A enzyme is replaced. It can effectively reduce the symptoms of FD while improving quality of life and patient survival when started in childhood, by reducing harmful Gb3 buildup in tissues and preventing progression to organ damage [18]. However, ERT will not reverse existing LVH in adults and does not appear to alter cardiac Gb3 deposition or fibrosis [19]; it is also unknown whether it will prevent further hypertrophy in adults. Furthermore, antiarrhythmic drugs alone are unsuitable for FD and will not prevent arrhythmias or sudden cardiac death in these patients [20]. Arrhythmias constitute an important cause of FD-related mortality, yet the effect of ERT on conduction abnormalities in FD is currently unclear. It is possible that early ERT may prevent significant fibrosis and arrhythmia later in life. Antiarrhythmic drugs may be used as an adjunct to anticoagulation or devices such as pacemakers or implantable cardioverter defibrillators to manage cardiac conduction disease in FD.

Importantly, due to cost, ERT is not readily available to patients in the state sector in South Africa or other African countries. A special application was made for our patient to receive ERT through the Sanofi Genzyme Humanitarian Program. Our patient is the first adult case to receive ERT for FD in the state sector in South Africa. This case emphasizes the important role genetics research can have in low-resource settings where diagnostic testing is not routinely available and sets an example of how research can be used to have direct clinical benefits for its participants, as without these research findings, the patient would likely never have received therapy tailored to his genetic condition. Furthermore, this illustrates the ethical responsibility to act on genetic results generated through research and ensure the appropriate follow-up for participants in whom a genetic result is found. In addition, it highlights the important contribution humanitarian treatment programs have in providing therapies that would otherwise not be available to patients with rare diseases living in lower-middle-income countries. Finally, work such as this emphasizes the need for genetic testing to become routine in all healthcare settings, where identification of rare genetic diseases can have profound impacts on patient management and family screening programs. Although currently unaffordable in many parts of the world, access to genetic testing should be prioritized when formulating healthcare policy and advocacy programs in resource-limited countries.

## Conclusions

In this study, we found pathogenic *GLA* variants in a South African family with HCM. These findings indicate that FD may represent an important, but underdiagnosed, contributor to HCM in South Africa. The diagnosis of FD, as in this family, should enable tailored therapy with ERT and clinical management with cascade genetic screening. Testing for *GLA* mutations may be warranted in other patients with similar unexplained LVH. This case also highlights the lack of routine genetic testing in resource-limited settings, as the FD in this patient was not diagnosed through the state sector but rather through a genetic research project. Consequently, FD is likely to be underreported in South Africa, with patients receiving inappropriate treatment, if any, for their condition. Genetic testing is not routine in South Africa due to costs and infrastructure challenges, and research such as the IMHOTEP study will be important first steps in understanding the genetic underpinnings of cardiovascular disease in South Africa. Future work should also include large-scale screening of *GLA* in HCM cohorts from lower-middle-income countries, as the contribution of FD in HCM is currently unknown.
